# DNA Virus Vectors for Vaccine Production in Plants: Spotlight on Geminiviruses

**DOI:** 10.3390/vaccines2030642

**Published:** 2014-08-05

**Authors:** Kathleen L. Hefferon

**Affiliations:** Cell and Systems Biology, University of Toronto, Toronto, Ontario M5S 2J7, Canada; E-Mail: kathleen.hefferon@utoronto.ca; Tel./Fax: +1-607-387-6304

**Keywords:** vaccine, virus expression vector, geminivirus, plant

## Abstract

Plants represent a safe, efficacious and inexpensive production platform by which to provide vaccines and other therapeutic proteins to the world’s poor. Plant virus expression vector technology has rapidly become one of the most popular methods to express pharmaceutical proteins in plants. This review discusses several of the state-of-the-art plant expression systems based upon geminiviruses that have been engineered for vaccine production. An overview of the advantages of these small, single-stranded DNA viruses is provided and comparisons are made with other virus expression systems. Advances in the design of several different geminivirus vectors are presented in this review, and examples of vaccines and other biologics generated from each are described.

## 1. Introduction

Over the past twenty years, great advances have been made in the construction and generation of plant-derived vaccines. Vaccines and other biopharmaceutical proteins produced from plants are safe, efficacious, and can be easily scaled up for mass production. Plant-made vaccines may provide a select advantage for specific demands that may not be readily addressed through conventional vaccine production. These range from providing inexpensive vaccines for the world’s poor who reside in developing countries to stockpiling vaccines against pandemic infectious diseases and even to generating plant made biologics for the field of personalized medicine [1,2].

Initially, vaccines and other pharmaceutical proteins were generated from transgenic plants, however, recent advances in plant virus molecular biology have yielded an alternative means of transiently expressing proteins through the use of virus expression vectors which are engineered to be delivery vehicles. Plant virus expression vectors offer many advantages for foreign protein expression over stably transformed plants; these include greater expression levels over a short period of time, the ability to generate proteins which may impede plant growth, as well as reduced biocontainment issues and related public perception concerns related to genetically modified crops. The deletion of virus movement and coat proteins disables plant to plant movement of virus expression vectors, and as a result, the possibility of pharmaceutical proteins being transmitted to weedy relatives is greatly reduced. Conversely, stable transgenic lines have the advantage of providing a permanent genetic resource which lacks significant variation in foreign protein expression and can be stored as seed [3].

Previously, cDNA constructs of plant virus expression vectors were introduced to plants, and the yield of pharmaceutical protein generated was determined in part by virus/host tissue specificity and by the lack of synchrony of virus produced during the course of a natural infection. Today however, efficiency of virus production has been substantially enhanced by inoculation via agroinfiltration. This involves infiltrating the leaves of a host plant with a syringe, for example, containing a suspension of Agrobacterium tumefaciens which harbours the virus vector. Vacuum infiltration of whole plants in a suspension of transformed *Agrobacterium* is also frequently used as a means to introduce virus vectors to plants [4].

Plant virus expression vectors which have been engineered to generate vaccines and other pharmaceutical proteins have predominantly been the positive-sense RNA viruses such as Tobacco mosaic virus, Potato virus X, Cucumber mosaic virus and Cowpea mosaic virus. Geminiviruses were among the first viruses to be considered as potential gene vectors but their use was limited because of the limitations on the size of insert tolerated. Recently, however, the geminiviruses have moved to the spotlight as highly effective expression vectors for vaccine production. The following review describes recent progress in geminivirus expression vector development, and their uses for the production of vaccines and other therapeutic proteins.

## 2. Concerning Geminiviruses

**Figure 1:**
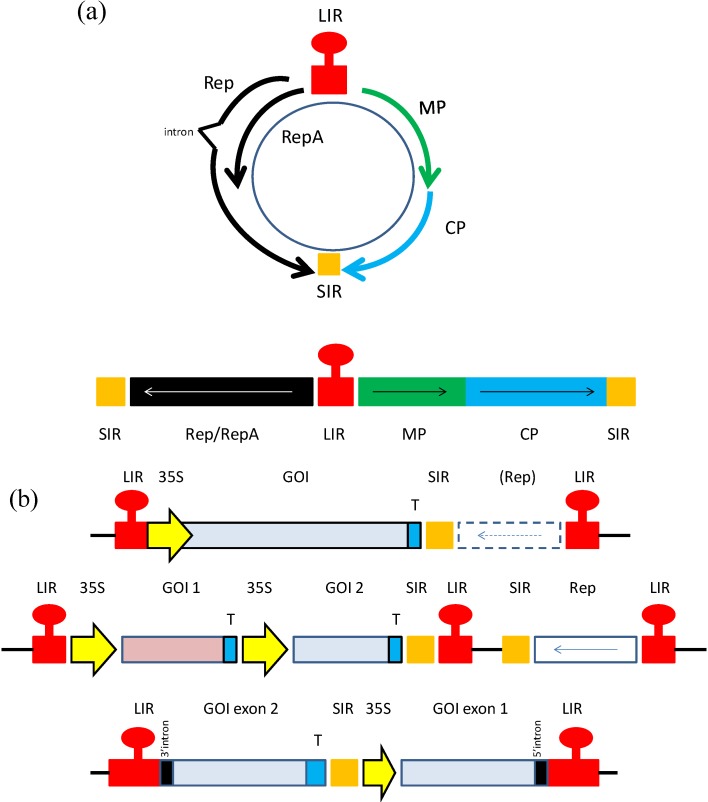
(**a**) Genomic organization of geminiviruses. An example of a mastrevirus is provided. Top; circular version, bottom; linearized version of genome. MP; movement protein, CP; coat protein, LIR and SIR; long and short intergenic regions; (**b**). Geminivirus expression constructs of past and present. Schematic representations of expression vector constructs based on geminiviruses. Top; example of an earlier expression vector. Middle; vector for co-expression of two different proteins, or complex multimeric protein such as monoclonal antibodies. Bottom; foreign gene is expressed in the form of two exons, and protein expression can only take place upon intron processing. In this case, Rep is expressed from an additional construct. GOI; gene of interest, 35S; 35S CaMV promoter, T; terminator. Hatched box; Rep gene could be present *in cis* in this construct, or expressed *in trans* from another construct. (middle construct sourced from [15], bottom construct sourced from [16]).

Geminiviruses, known for their twinned capsid morphology, have been employed as production platforms for the generation of both pharmaceutical and industrial proteins. The small single stranded circular genome ranges from 2.5–3.2 kb in length. This genome is ambisense and both monopartite and bipartite versions of the genome exist. Although the family of geminiviruses is large and includes multiple genera, all geminiviruses in general encode a movement protein, a coat protein and a replication initiator protein which is required for rolling circle replication (RCR) of the virus. The organization of *cis*-acting elements also possess several features in common throughout geminiviruses. Whether monopartite or bipartite, all geminiviruses contain an intergenic region, which holds a stem loop structure, located within the origin of replication, as well as divergent promoter elements responsible for sense and complementary-sense gene expression (Figure 1a) [5,6].

It has been suggested that geminiviruses originate from a common ancestor along with other small, circular, single-stranded DNA viruses of related genomes, including nanoviruses, circoviruses and cycloviruses. This group of viruses infect a diverse array of organisms, ranging from plants and mammals to birds, fish and insects [7,8]. The close phylogenetic homology between the Rep protein of geminiviruses and some phytobacteria, for example, have led to the hypothesis that geminiviruses have evolved from a phytoplasma plasmid or other episomal replicon [9,10,11]. Similarly, many Rep-like fossil sequences have been found in a broad spectrum of eukaryotic genomes [12,13,14].

Geminiviruses replicate via a rolling circle mechanism and use a replication initiator protein, Rep, that initiates replication at a highly conserved stem-loop structure located between two major open reading frames of the genome. The replication strategy of geminiviruses has been reviewed extensively and will be only briefly described here [17,18]. Following infection, the virus particle enters the nucleus via a nuclear localization signal on the coat protein, and after release of the viral single-stranded DNA, host cell DNA polymerase I and components of DNA repair machinery synthesize a complementary strand to generate a double-stranded DNA intermediate. Upon association with nucleosomes to form a “minichromosome” the replication initiator protein (Rep) is expressed from the leftward promoter and then initiates rolling circle replication by nicking the virion strand of the dsDNA template at a highly conserved nonanucleotide sequence located within the origin of replication. A shorter version of Rep, known as RepA, is also expressed from this open reading frame and is responsible for changing the environment of the infected plant cell into one that is more permissive for replication [18]. Then, using the host cell replication machinery, Rep generates multiple copies of virion-sense strand ssDNA using the complementary-sense strand as a template. The virion-sense strand become displaced from the template strand, then is nicked and religated by Rep to be released as multiple copies of circular ssDNAs, which can either undergo RCR or become packaged into mature virions during the late stage of infection [17,18].

This mechanism of replication is highly effective and can result in the expression of tens of thousands of copies of the viral genome per cell, making geminiviruses robust vehicles for high levels of foreign gene expression. Geminiviruses have other advantages as well. For example, upon complementary strand DNA synthesis, the viral dsDNA genome associates with nucleosomes and forms minichromosomes which remain in an unmethylated, transcriptionally active state [19,20]. The fact that the geminivirus genome is represented in the form of a single-stranded DNA and undergoes Rep-mediated unmethylated replication makes it difficult for the host methylation machinery to actively repress unwanted viral transcription of virus directed gene products within the nucleus [21,22]. Moreover, many different members of the geminivirus family possess a variety of gene silencing evasion strategies. Geminiviruses have evolved different suppressor proteins which interfere with potential host cell transcriptional silencing events [23]. These include AC2/AL2/C2/L2 homologs, which are encoded by *Begomoviruses* and *Curtoviruses*, and betaC1, which is encoded by betasatellites that are associated with a number of begomoviruses [23,24].

## 3. Geminiviruses as Expression Vectors

### 3.1. Early Expression Vector Studies

Since geminiviruses can accumulate to extremely high copy numbers in infected cells, resulting in tremendous levels of gene expression, for many years they have been investigated as potential expression vectors for the production of vaccines and other biopharmaceutical proteins. Initial studies involved the substitution of the virus gene encoding the capsid protein (CP) for a reporter gene such as GFP or GUS. Removal of the capsid protein open reading frame maintained the size limitation of the virus genome and also disabled the ability of the virus to become encapsidated or move from cell to cell, resulting in a contained infection (Figure 1b). Geminiviruses such as the mastreviruses Maize streak virus and Wheat dwarf virus were demonstrated to express reporter proteins in this fashion to extremely high levels in plant cells [25,26,27,28,29,30,31]. From these initial results, new constructs were made based on more deconstructed geminivirus sequence strategies. For example, the gene encoding Rep of the mastrevirus Bean yellow dwarf virus was placed under Caulifower mosaic virus (CaMV) 35S constitutive independent promoter control and the *cis*-acting elements required for replication were subcloned along with a reporter gene into a separate plasmid [32]. This strategy enabled Rep to initiate replication and gene expression *in trans.* This vector was later demonstrated to express the vaccine protein Staphylococcus enterotoxin B in a tobacco cell line via particle bombardment at levels up to 50-fold higher in the presence of Rep rather than with the expression construct alone [33]. The BeYDV based vector was also used to express reporter genes at high levels under either developmental or alcohol-inducible promoters [34,35]. The implications of these results are significant; with the use of inducible or developmental promoters to regulate Rep expression, virus replication and foreign gene expression can be initiated when so desired. This helps to avoid toxicity issues in the plant and can enable the protein of interest to accumulate in only specific plant tissues, such as seed, for example.

Geminivirus vector design has grown exponentially in sophistication and examples of the use of geminiviruses are beginning to flood the literature. The next section discusses several of the vectors in use today and provides examples of their use. These examples are summarized in Table 1. 

**Table 1:** Geminiviruses vectors listed in this review.

Geminivirus	Function of Vector	Reference
Wheat Dwarf Virus Expression Vector [23] Maize streak virus (MSV)	Expression vector	[26,27]
Bean yellow dwarf virus (BeYDV)	vaccines, gene silencing, DNA repair	[35,36,37]
Mild stain of BeYDV	Vaccine production	[38,39]
Beet curly top virus (BCTV)	Vaccine production	[40,41]
Tobacco yellow dwarf virus (TYDV)	Vaccine/industrial protein production	[16,42]
5Tobacco curly shoot virus (TbCSV)	Gene silencing	[43]
Cabbage leaf-curl virus (CaLCuV)	Gene silencing	[44]
African cassava mosaic virus (ACMV)	Gene silencing	[45]
Ageratum yellow vein virus (AYVV)	Expression Vector	[46]
Abutilon mosaic vírus (AbMV)	Gene silencing	[47]
Cotton leaf curl Multan betasatellite (CLCuMB)	Gene silencing	[48]

### 3.2. Next Generation Geminivirus Expression Vectors

#### 3.2.1. BeYDV Vaccine Vectors

The BeYDV-based vectors generated today are more refined both in design and flexibility [49]. These second generation vectors have been provided with new attributes such as open reading frames that encode suppressors of gene silencing, for example, or that can co-express several open reading frames in tandem, so that more complex proteins such as antibodies can be generated from a single construct (Figure 1b) [35]. These vectors are capable of increasing yields of foreign protein as great as 1 mg/g fresh leaf tissue [36]. The challenge now is to make these vectors amenable to large scale production and to develop production strategies that comply with Good Manufacturing Practice (cGMP) and can readily be provided it in a form that can be administered to patients.

Besides SEB, a number of vaccine and therapeutic proteins have been expressed using the BeYDV vector series and are listed in Table 2. One of the great triumphs of this expression system has been the ability to express in plants the epitopes to a number of serious pathogens on the surface of virus-like particles. For example, Norwalk virus capsid protein (NVCP) expressed in plants using the BeYDV geminivirus system can readily self-assemble into virus-like particles within 4 days post agro-inoculation [36]. Similar results were obtained with Hepatitis B core antigen (HBcAg) [34]. Further work has included the generation of a vaccine for West Nile virus as well as a monoclonal antibody for Ebola virus [34,35]. Vaccine proteins have been expressed in both tobacco as well as in lettuce, a crop plant that is readily available to produce en masse, but lacks the nicotine and harmful phenolics present in tobacco that require removal prior to manufacturing. For example, NVCP accumulated to levels of ~0.2 mg/g leaf fresh weight in lettuce [36]. The VLPs derived from NVCP were highly similar to those produced in insect cells and could be readily purified using a two-step process. Moreover, monoclonal antibodies to Ebola virus (EV) GP1 protein and West Nile Virus (WNV) E protein were generated from a single vector replicon and accumulated at levels of ~0.23 to 0.27 mg/g leaf fresh weight in lettuce plants [37]. In all of the above cases, expression of these biologics was greatest at 4 days post-inoculation, further demonstrating the rapid nature of this expression platform. The monoclonal antibodies were demonstrated to bind specifically to their antigens by ELISA and flow cytometry assays. The WNV Mab was also shown to be biologically active using a focus reduction neutralization assay [36].

**Table 2:** Vaccines and other therapeutic proteins produced from geminivirus vectors.

Therapeutic Protein	Vector Used	Host Plant	Expression Level	Immunogenicity Tested	Reference
SEB	BeYDV	*N. bethamiana*	n/a *	yes	[31]
Norwalk Virus VLPs	BeYDV	Tobacco, lettuce	0.34 mg/g LFW **	yes	[36]
HBVcAg	BeYDV	*N. benthamiana*	0.8 mg/g LFW	no	[34]
WNV E protein Mab	BeYDV	Tobacco, lettuce	0.23–0.27 mg/g LFW	yes	[35]
Ebola Virus GP1Mab	BeYDV	Tobacco, lettuce	0.23–0.27 g/g LFW	yes	[35]
HPV-1 L1 protein	BeYDV, mild strain	*N. benthamiana*	n/a	no	[38]
HIV-1 type C p24	BeYDV, mild strain	*N. benthamiana*	n/a	no	[38]
HAV VP1	BCTV	*N. benthamiana*	n/a	no	[41]
vitronectin	TYDV	*N. benthamiana*	2.3% TSP ***	n/a	[42]

* n/a: not applicable; ** LFW: leaf fresh weight; *** TSP: total soluble protein.

Another version of a BeYDV expression vector known as pRIC designed by Ed Rybicki’s research group was used to generate a candidate vaccine for human papillomavirus-16 (HPV-16) based on the capsid protein L1, as well as a vaccine for HIV-1 type C p24 antigen based on the Gag protein [38,39]. This vector was developed from a mild strain of BeYDV that is replicationally released into plant cells from a Ti plasmid through agro-infection. In this case, all Rep open reading frames were present *in cis* rather than *in trans* on the construct, and the coat protein and movement protein genes were replaced by an expression cassette derived from pTRAc, a nonreplicating *A. tumefaciens* vector. Using as examples the reporter enhanced green fluorescent protein EGFP as well as the subunit vaccine antigens for HPV and HIV listed above, the authors improved replication efficiency in *N. benthamiana* by two orders of magnitude and increased protein expression with the pRIC vector by 3–7 fold for EGFP and HIV-1, and 50% for HPV-16. This improved expression vector system therefore offers increased yields of vaccine protein, and thus presents a step forward toward mitigating a significant hurdle in plant molecular pharming [38,39].

#### 3.2.2. BCTV Vaccine Vector

Another geminivirus known as Beet curly top virus (BCTV) has been engineered as a vaccine expression vector. This vector was constructed by substituting the Cassava vein mosaic virus (CsVMV) promoter in place of the CAMV 35S promoter. Reporter gene expression increased by 320% at the RNA level and protein expression up to 240% when the P19 suppressor of gene silencing was supplied [40]. The capsid protein to Hepatitis A virus (HAV VP1) was fused to the Fc antibody fragment and expressed in *N. benthamiana*. Recombinant HAV VP1-Fc purified by affinity chromatography was able to elicit a serum IgG response after intraperitoneal immunization. IFN-γ and IL-4 levels were also shown to increase upon immunization (Table 2) [41].

#### 3.2.3. TYDV Vaccine Vector

Dugdale *et al.*, (2013), have developed a technology based on the mastrevirus Tobacco yellow dwarf virus (TYDV) that offers an interesting new twist on previous geminivirus expression vector constructs (Figure 1b) [16,42]. This TYDV system is composed of a two-expression cassette system; one expression cassette encodes Rep/RepA under the control of the AlcA:AlcR promoter and the second expression cassette contains the gene of interest activated under the control of an ethanol inducible promoter, which can be activated by the simple application of an ethanol spray. The gene of interest is placed into the INPACT (In Plant Activation) cassette in a manner that it is split into two parts, divided by a synthetic intron. In this way, the gene of interest can only be expressed from replicons that are produced during activation of the geminivirus sequences and processed to remove the intron. These sequences are in turn activated only upon the presence of ethanol. High amounts (2.5% of TSP) of expression of the protein of interest were recorded, and the use of the alcohol-inducible promoter enables expression to be controlled in a temporal, spatial and dose-dependent manner (Table 2). Furthermore, this system has proven to be adaptable to many different host plant species, offering a select advantage over many other plant virus expression systems which are currently available. As a proof of concept, the therapeutic protein vitronectin was produced and easily purified from leaves harboring this virus expression system and sprayed with 1% ethanol.

## 4. Other Geminivirus Vectors and Their Uses

Besides their potential in the pharmaceutical field, geminivirus expression vectors, with their broad host range and ease of use, have also been used for functional genomics studies [49]. For example, geminiviruses have been employed as virus-induced gene silencing (VIGS) vectors, by downregulating the expression of a specific gene and determining its function in the resulting phenotype. Examples of geminiviruses that have been modified for use in this manner include tobacco curly shoot virus (TbCSV), cabbage leaf-curl virus (CaLCuV), African cassava mosaic virus (ACMV), Ageratum yellow vein virus (AYVV). and Abutilon mosaic virus (AbMV) [43,44,45,46,47].

Vaccine expression vectors based on other geminiviruses are also under development. For example, Cotton leaf curl Multan betasatellite (CLCuMB) requires the presence of the helper viruses tomato leaf curl virus (ToLCV) or beet severe curly top virus (BSCTV). An expression vector was engineered from this betasatellite by replacing the βC1 ORF with segments of either the CaMV 35S or the petunia ChsA promoter. The authors showed that they could silence reporter gene activities in transgenic plants expressing by 35SGUS and in nontransgenic petunia plants expressing ChsA by supplying this betasatellite in the presence of helper virus [48].

Recently, the Bean yellow dwarf geminivirus vector has been used to deliver sequence-specific nucleases and DNA repair templates to facilitate homologous repair of double-stranded breaks within plant sequences [50,51]. Repair efficiency was in fact improved using the geminivirus as a delivery vehicle compared to other delivery methods; this may possibly be due to the ability of Rep A to alter the cell cycle to become more permissible for DNA repair machinery to be functioning. Moreover, calli and plantlets which harbour a precise change in sequence can be regenerated from plant tissue that is agroinoculated with the geminivirus vector repair system. Geminiviruses can therefore be used to quickly generate specific genome modifications, an important step in genome engineering for crop improvement [50,51].

## 5. Conclusions

Plant virus expression vectors have been engineered to function as rapid, inexpensive and robust platforms for vaccine production. The implications of this and other technologies related to molecular pharming in plants are substantial. Geminivirus vectors offer select advantages over plant RNA virus expression vectors, for example, they are able to function in a much broader range of plants, and thus provide more choices of the production system to be used. Geminiviruses lack the stability issues that their RNA virus vector counterparts experience [52]. Geminiviruses also seem to have developed several means to evade gene silencing, an issue that hinders many plant expression systems. The ability to express several proteins in tandem and at comparable levels from a single construct could provide added value over other virus vectors. As research and development progresses, the uses of geminiviruses will amplify and become even more elaborate in design and function. In conclusion, geminivirus expression vectors represent a state-of-the art method by which to generate vaccines and other therapeutic proteins from plants.
